# The Efficacy of Acupuncture on Anthropometric Measures and the Biochemical Markers for Metabolic Syndrome: A Randomized Controlled Pilot Study

**DOI:** 10.1155/2017/8598210

**Published:** 2017-10-31

**Authors:** Mingjuan Han, Yuxiu Sun, Wei Su, Shixi Huang, Sinuo Li, Mingyue Gao, Wenyan Wang, Fang Wang, Zhaohui Fang, Hong Zhao

**Affiliations:** ^1^Institute of Acupuncture and Moxibustion, China Academy of Chinese Medical Sciences, Beijing 100700, China; ^2^Being University of Chinese Medicine, Beijing 100029, China; ^3^Beijing First Hospital of Integrated Chinese and Western Medicine, Beijing 100026, China; ^4^Guang'anmen Hospital, China Academy of Chinese Medical Sciences, Beijing 100053, China; ^5^Wangjing Hospital, Chinese Academy of Chinese Medicines, Beijing 100102, China; ^6^First Affiliated Hospital, Anhui University of Traditional Chinese Medicine, Hefei, Anhui Province 230031, China

## Abstract

**Background:**

Many previous studies have shown the potential therapeutic effect of acupuncture for metabolic syndrome (MetS). However, most of these studies were limited by short durations of observation and a lack of sham acupuncture as control. We designed a randomized controlled trial, used sham acupuncture as the control, and evaluated the efficacy over 12 weeks of treatment and 12 weeks of follow-up.

**Methods/Design:**

The study was designed as a multicentre, parallel-group, randomized, double-blinded trial. 40 patients were randomly assigned to two groups: treatment group (treated with acupuncture) and control group (treated with sham acupuncture). Outcomes were measured at 4, 8, and 12 weeks and 3 months after treatment.

**Results:**

33 participants (17 in acupuncture group and 16 in control group) completed the treatment and the follow-up. Decreases from baseline in mean waist circumference (WC) and weight at the end of treatment were 4.85 cm (95% CI [2.405,5.595]) and 4.00 kg (95% CI [1.6208,4.4498]) in acupuncture group and 1.62 cm and 1.64 kg in control group (*P* < 0.01). The changes in mean glycosylated haemoglobin (HbA1c), triglycerides (TG), total cholesterol (TC), and blood pressure in acupuncture group were greater than the changes in control group (*P* < 0.05, *P* < 0.01).

**Conclusion:**

Acupuncture decreases WC, HC, HbA1c, TG, and TC values and blood pressure in MetS.

## 1. Introduction

Metabolic syndrome (MetS) is a cluster of metabolic symptoms including abdominal obesity, hypertension, dyslipidaemia, and insulin resistance (IR) [1]. Several frequently used definitions of MetS currently exist, including definitions proposed by the World Health Organization (WHO) [2], the National Cholesterol Education Program Adult Treatment Panel III (NCEP ATP III) [3], the International Diabetes Federation (IDF) [4], the International Diabetes Federation (IDF), and the American Heart Association/National Heart, Lung, and Blood Institute (AHA/NHLBI) [5]. They show different reference values and variations concerning the metabolic parameters associated with the syndrome.

MetS has become an increasing problem in our society. The prevalence of MetS has increased as a result of the increasing prevalence of obesity and sedentary lifestyles [6]. In the United States, approximately 25% of adults have been diagnosed with metabolic syndrome [7]. According to the results of epidemiological studies, the prevalence of MetS in China is 13.3% [8].

A systematic review showed that MetS was associated with a high risk for diabetes, cardiovascular disease (CVD), CVD mortality, and stroke [9]. Therefore, it is very important to prevent, treat, and control MetS. Lifestyle modification is the essential strategy for MetS prevention and treatment and includes weight control, diet management, exercise, and smoking cessation [10, 11]. Drug therapy includes statins, angiotensin-converting enzyme (ACE) inhibitors, angiotensin II receptor blockers (ARBs), and metformin to target dyslipoproteinemia, hypertension, and dysglycaemia, respectively [12].

Despite the many treatment methods for MetS, it remains a difficult public health issue. Acupuncture, with thousands of years of history, may have a potential beneficial effect in this disease. In the ancient literature, records suggest acupuncture for the treatment of obesity and metabolism. Both experimental and clinical studies have shown that acupuncture can not only reduce weight but can also lower plasma glucose levels and improve insulin resistance [13, 14]. However, clinical evidence for acupuncture in the treatment of MetS is insufficient. Therefore, we designed a pilot randomized controlled study to evaluate the efficacy of acupuncture in the treatment of metabolic syndrome and to explore the feasibility of this clinical trial.

## 2. Methods

### 2.1. Research Design

We conducted a prospective, randomized, double-blinded controlled trial. We used a computer program to determine the random numbers. Numbers were put into opaque envelopes. Patients were randomly assigned to the treatment group or the control group. The treatment group received acupuncture and the control group received sham acupuncture. Both the investigators and the participants were unaware of the treatment allocation.

### 2.2. Participants

Patients were recruited from Guang'anmen Hospital of Chinese Academy of Chinese Medical Science (CACMS). Participants were included if they met the diagnostic criteria for MetS based on International Diabetes Federation (IDF) and American Heart Association/National Heart, Lung, and Blood Institute (AHA/NHLBI) definitions [5]. 3 or more of the following 5 risk factors are defined as MetS: (1) elevated waist circumference, WC ≥ 90 cm for male and WC ≥ 80 cm for female; (2) elevated triglycerides (drug treatment for elevated triglycerides is an alternate indicator), TG ≥ 150 mg/dL (1.7 mmol/L); (3) reduced HDL-C (drug treatment for reduced HDL-C is an alternate indicator), HDL-C < 40 mg/dL (1.0 mmol/L) in males and HDL-C < 50 mg/dL (1.3 mmol/L) in females; (4) elevation of blood pressure (antihypertensive drug treatment in a patient with a history of hypertension is an alternate indicator), systolic ≥ 130 and/or diastolic ≥ 85 mm Hg; (5) elevated fasting glucose (drug treatment of elevated glucose is an alternate indicator), FBG ≥ 100 mg/dL. Patients were between 18 and 60 years of age, had not participated in diet management or other drug therapy for one year, agreed to participate in this study, and were able to participate in 3 months of therapy and 6 months of follow-up. Patients were excluded if they had any of the following conditions: other causes of obesity, such as adrenal cortex hyperfunction, hypothyroidism, insulin cell tumour, or polycystic ovary syndrome; chronic diseases requiring the long-term use of hormones; severe coronary heart disease; stroke disorder; kidney disease; infectious diseases; mental illness; fear of acupuncture treatment; or an inability to adhere to treatment. All participants signed informed consent.

### 2.3. Intervention

We developed the interventions according to the ancient literature and experts' clinical experience. Four licensed acupuncturists with 1 to 2 years of experience provided treatment. Treatment consisted of 20–24 sessions for a total of 12 weeks (3 sessions during each of the first 4 weeks and 1-2 sessions during each of the remaining 8 weeks). Each session was 30 minutes. Disposable stainless steel needles (Suzhou Medical Appliance) of 0.25 × 25 mm and 0.25 × 40 mm in size were used. The participants were positioned on their backs during treatment. After routine sterilization, the needles were inserted into the skin.

The participants in the treatment group received acupuncture at the bilateral acupoints of Tianshu (ST 25), Daheng (SP 15), Daimai (GB 26), Liangmen (ST 21), Zusanli (ST 36), Qihai (CV 5), Zhongwan (CV 12), and Xiawan (CV 10) on the ventral middle line of the body. The sham acupoints are all located 0.5 cm next to the acupoints.

All acupoints were determined according to the WHO International Standard Acupuncture Points. The needles were inserted into the acupoints perpendicularly. The depths of needle penetration ranged from 1 to 3 cm. The depth of needle penetration at each acupuncture point in each participant was different according to the degree of obesity. Then, the acupuncture physicians carried out twirling manipulation until the patient and the physician felt the sensation of* qi*. The needles were retained for 30 minutes after the onset of* qi*.

The participants in the control group received shallow needling at bilateral sham Tianshu (ST 25), sham Daheng (SP 15), sham Daimai (GB 26), sham Liangmen (ST 21), sham Zusanli (ST 36), sham Qihai (CV 5), sham Zhongwan (CV 12), and sham Xiawan (CV 10). These sham nonacupoints were located 0.5 cm next to ST 25, SP 15, GB 26, ST 21, ST 36, CV 5, CV 12, and CV 10, respectively. Specifically, the needles were inserted approximately 2 to 5 mm into these sham nonacupoints without any manipulation. The procedure for acupuncture therapy was standardized by training in advance and two researchers with attending physician titles reviewed the therapy on a regular basis to ensure that the acupuncture operations conformed to the requirements of the study design (see Supplement Table in the Supplementary Material available online at https://doi.org/10.1155/2017/8598210).

*Concomitant Care and Intervention*. During the treatment and follow-up periods, the participants were prohibited from taking medications such as weight loss drugs, antihypertensive drugs, and glucose-lowering and lipid-lowering drugs. The participants were required to change their lifestyles, exercise, limit carbohydrates, and abstain from alcohol. The type and frequency of exercise and changes in diet were recorded using WeChat tools.

### 2.4. Outcomes

Anthropometric measures were assessed by two assistants at baseline, every 2 weeks during the treatment, and follow-up periods (12 weeks after intervention). Height and weight were measured by an electronic scale and a wall-mounted stadiometer. Waist circumference (WC) and hip circumference (HC) were measured in a separate room using a tape measure. Each measurement was taken twice and the average values were recorded. In addition, the waist-to-hip ratio (WHR) and the body mass index (BMI) were calculated according to the following formula: WHR = waist circumference (cm)/hip circumference (cm); BMI = weight (kg)/height^2^ (m^2^).

Blood pressure (BP) measurements included systolic blood pressure (SBP) and diastolic blood pressure (DBP), which were measured by two assistants using a mercury sphygmomanometer device at baseline, every 2 weeks during the treatment, and follow-up periods (12 weeks after intervention). Fasting blood glucose (FBG), glycosylated haemoglobin (HbA1c), triglycerides (TG), total cholesterol (TC), high density lipoprotein (HDL), low density lipoprotein (LDL), and uric acid (UA) were measured at baseline and 12 weeks after treatment. 10 ml venous blood samples were taken from each participant. The samples were tested at the laboratory in the Guang'anmen Hospital for assessment of the above biochemical indexes.

The primary outcomes included the changes from baseline in mean WC and HC every two weeks during weeks 1 to 12. The secondary outcomes included the changes from baseline in mean weight, BMI, WHR, SBP, DBP, FBG, HbA1c, TG, TC, HDL, LDL, and UA during the treatment period and follow-up periods.

Adverse events, such as pain, subcutaneous bleeding, and needle sticks, were observed and managed by the acupuncturists.

### 2.5. Statistical Analysis

The statistical analysis was performed with SPSS 19.0 software (SPSS Inc., Chicago, IL, USA) in the Clinical Evaluation Center of CACMS. Descriptive statistics were used to compare the baseline measures and the patient characteristics between the two groups. For the primary and secondary outcome measures, a two-sample *t*-test was used to compare the differences between the two groups at baseline and at the end of treatment (*P* < 0.05 will be considered statistically significant). The paired *t*-test was used to compare the differences in the primary and secondary outcomes before and after treatment.

## 3. Results

### 3.1. Participant Flow

**Figure 1:**
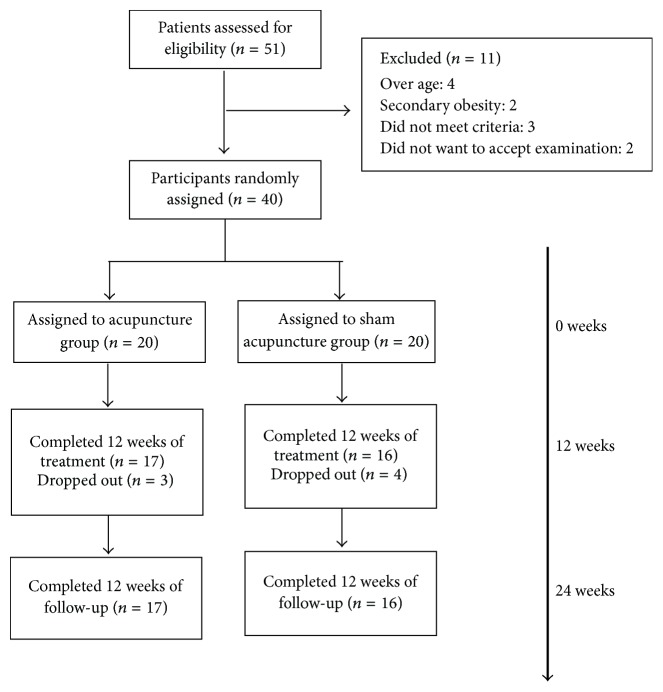
Trial flow diagram.

From 10 May 2015 to 31 December 2015, 51 patients were screened and 40 participants were enrolled, including 20 subjects in the acupuncture group and 20 subjects in the sham acupuncture group. 33 participants (17 in the acupuncture group and 16 in the sham acupuncture group) completed the treatment and the follow-up. 3 patients in the acupuncture group withdrew because of unwanted pregnancy, business, or other treatments. 4 patients in the sham acupuncture withdrew because of other obligations or invalid treatment. During the entire session, 9 participants (5 in the acupuncture group and 4 in the sham acupuncture group) missed the metabolic index but had simple parameters of body fat (Figure 1).

No significant differences were observed between the two groups in sex, age, simple parameters of body fat, or the biochemical markers at baseline (Table 1).

### 3.2. Anthropometric Measures

**Figure 2:**
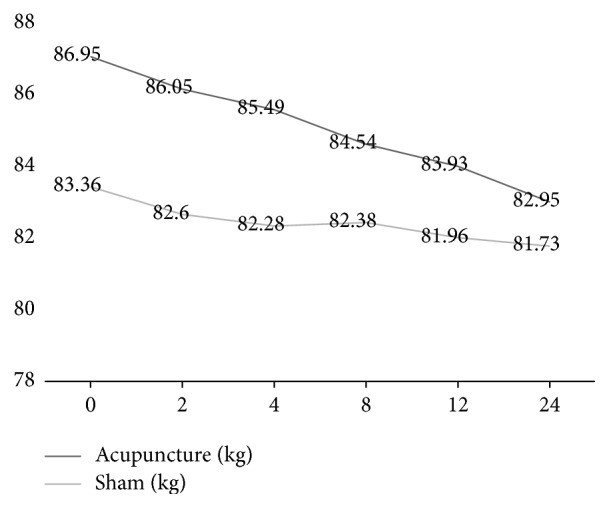
Variety of weight of participants treated with acupuncture and sham acupuncture.

**Figure 3:**
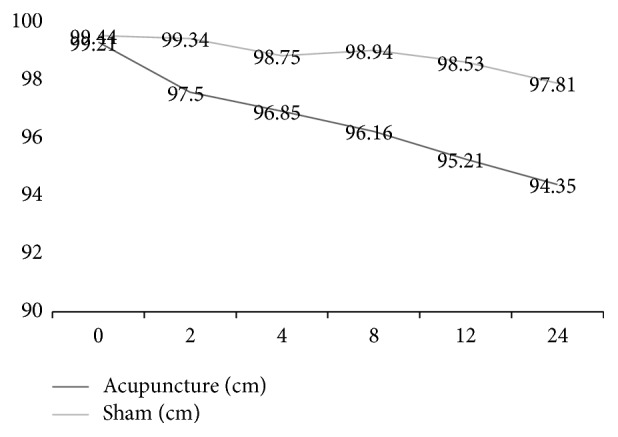
Variety of WC of participants treated with acupuncture and sham acupuncture.

**Figure 4:**
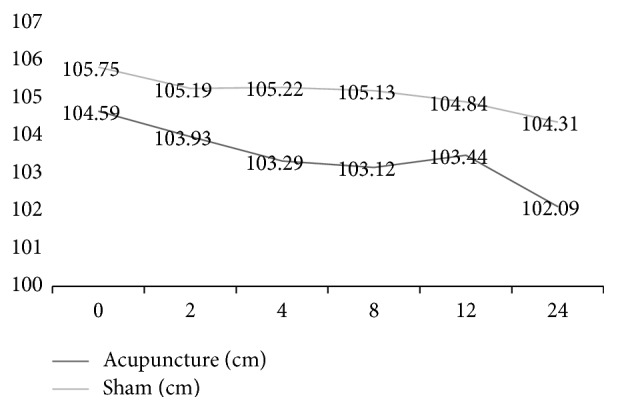
Variety of HC of participants treated with acupuncture and sham acupuncture.

At the end of the 12-week treatment, the decreases from baseline in mean WC, weight, BMI, and WHR were 4.85 cm, 4 kg, 1.37, and 0.02 in the acupuncture group and 1.62 cm, 1.64 kg, 0.64, and 0.01 in the sham acupuncture group, respectively, with significant differences between the two groups (*P* = 0.003, *P* = 0.011, *P* = 0.010, *P* = 0.004). No significant difference in the mean change in HC was observed between the two groups (*P* = 0.118) (Figures 2, 3, and 4 and Table 2).

At the 3-month follow-up, the decreases from baseline in mean WC, weight, BMI, and WHR were 2.5 cm, 0.92 kg, 4.85, and 0.01 in the acupuncture group and 1.44 cm, 0.24 kg, 0.09, and 0.01 in the sham acupuncture group, respectively, with significant differences between the two groups (*P* = 0.002, *P* = 0.007, *P* = 0007, *P* = 0.010). No significant difference was observed between the two groups in the mean change in HC (*P* = 0.054).

The within-group analysis showed that WC, HC, weight, and BMI were significantly reduced in the acupuncture group (*P* ≤ 0.001, *P* = 0.035, *P* ≤ 0.001, *P* = 0.002) and in the sham acupuncture group (*P* = 0.047, *P* = 0.034, *P* = 0.135, *P* = 0.304) after 12 weeks of treatment.

The within-group analysis showed that WC, HC, weight, and BMI were significantly reduced in the acupuncture group (*P* ≤ 0.001, *P* = 0.002, *P* = 0.001, *P* = 0.001) and in the sham acupuncture group (*P* = 0.101, *P* = 0.016, *P* = 0.144, *P* = 0.198) at the 3-month follow-up.

### 3.3. The Biochemical Markers

At the end of 12 weeks of treatment, the decreases from baseline in mean HbA1c, TC, SBP, and DBP were 0.1, 8, 10, and 10 in the acupuncture group and −0.1, 4, 4, and 5 in the sham acupuncture group, respectively, with differences between the two groups (*P* = 0.008, *P* = 0.019, *P* = 0.010, *P* ≤ 0.001). No differences were observed between the groups in the mean changes in FBG, TG, HDL, LDL, or UA (*P* = 0.217, *P* = 0.046, *P* = 0.814, *P* = 0.477, *P* = 0.059) (Table 3).

The within-group analysis showed that UA, SBP, and DBP were significantly reduced in the acupuncture group (*P* = 0.036, *P* ≤ 0.001, *P* ≤ 0.001) and in the sham acupuncture group (*P* = 0.319, *P* = 0.168, *P* = 0.071) after 12 weeks of treatment.

### 3.4. Safety Evaluation

In the acupuncture group, 5 cases of bleeding and 1 case of blood stasis occurred, but these patients recovered after two weeks and experienced no residual discomfort. No adverse events occurred.

## 4. Discussion

MetS is characterized by a variety of abnormal metabolic components. Domestic and foreign research shows that abdominal obesity plays an important role in MetS and is associated with other components of MetS. The Chinese population shows more abdominal obesity (high waist circumference) [15, 16]. For MetS, the treatment for abdominal obesity is particularly important. Other studies have shown that the body can benefit from weight loss, even if it is only 5%–10% of the total body weight [17]. Weight loss can improve obesity-related diseases such as hypertension and abnormal lipid metabolism, reduce the serum insulin level, and improve insulin sensitivity. Acupuncture has certain advantages in the prevention and control of obesity, especially abdominal obesity [18]. However, the effects of acupuncture for the treatment of MetS on body weight and abdominal obesity and their mutual relationship are not clear. Our study showed that decreases from baseline in mean WC and weight at the end of treatment were 4.85 cm (95% CI [2.405,5.595]) and 4.00 kg (95% CI [1.6208,4.4498]), respectively, in the acupuncture group and 1.62 cm and 1.64 kg in the sham acupuncture group, respectively (*P* < 0.01). Starting from the second week of treatment, at every measurement time point, the decreases from baseline in mean WC in the acupuncture group were greater than in sham acupuncture group. However, in mean weight and BMI, the changes showed statistically significant differences between the two groups after 8 weeks of treatment. These results indicated that acupuncture may mainly reduce WC in the first two to four weeks by improving the body fat distribution and may reduce weight after 8 weeks of treatment.

In our study, we selected regular acupoints to treat obesity and abdominal obesity and to attach clearing damp tong qi and hysteresis effects. We used single acupuncture and no other acupuncture methods such as electric acupuncture or cupping. Our observations showed that the participants in the acupuncture group experienced a mean reduction in weight of 4.00 kg and a mean reduction of 4.85 cm in WC, which were slightly better than the results of other single acupuncture intervention clinical trials. The results of one clinical trial showed that acupuncture reduced weight and WC after 8 weeks with filiform acupuncture treatment. Another study suggested that electric acupuncture may have a better effect and that cupping and acupuncture point bury line therapy may also enhance the effectiveness [19]. These results may indicate that a multifactor intervention for treatment is superior to the simple acupuncture treatment.

In addition, HC did not show any statistically significant changes, perhaps because we selected acupuncture points mainly in the waist and lower limbs and not in the hip. Further research should include hip points to potentially improve the curative effect of acupuncture in the hip area.

Compared with the sham acupuncture group, HbA1c, SBP, and DBP in the acupuncture group decreased significantly more (*P* < 0.01). This indicated that acupuncture may prevent and treat MetS by reducing a patient's blood pressure and controlling increases in blood sugar. In our study, FBG, HbA1c, and TG in the sham acupuncture increased. We suspect that these results may be related to loose quality control among the participants and small sample sizes. We found that patients with MetS lack attention to lifestyle management and metabolic index monitoring. During the entire study, we could not implement strict lifestyle management among the participants. In future clinical research for MetS, participants' lifestyles should be strictly managed by research supervisors.

Our study has some limitations. First, the number of lost participants was too great. We should improve the management of the participants' lifestyles and their compliance. Second, our sample size was small. We expected that the study could provide a basis for a sample size calculation for further research. Third, the number of acupoints in the intervention was insufficient. We will improve the intervention plan in further research. These limitations will be explored to provide important lessons for future research.

From this pilot study, the results indicate that acupuncture decreases WC, HC, HbA1c, TG, TC, and blood pressure in MetS. Rigorous clinical studies are needed to further clarify the efficacy and the long-term effects of acupuncture.

## Supplementary Material

Acupoints location and manipulation.

## Figures and Tables

**Table 1:** Baseline characteristics of the randomly assigned participants.

Variable	Acupuncture (*n* = 17)	Sham acupuncture (*n* = 16)	*P* value
Age, mean (SD), y	37.18 (9.36)	38.5 (13.22)	0.482
Male *n*, (%)	7 (41.18%)	5 (31.25%)	0.723
BMI, mean (SD), (kg/m^2^)	30.77 (3.83)	30.89 (5.15)	0.651
WC, mean (SD), (cm)	99.21 (9.62)	99.44 (11.3)	0.718
FBG, mean (SD), (mmol/dL)	5.76 (1.75)	7.22 (3.06)	0.538
HbA1c, mean (SD), (%)	5.59 (0.54)	6.35 (1.53)	0.069
TG, mean (SD), (mg/dL)	2.19 (2.37)	1.86 (0.89)	0.319
TC, mean (SD), (mg/dL)	5.08 (1.07)	5.23 (1.09)	0.719
HDL, mean (SD), (mg/dL)	1.26 (0.3)	1.33 (0.26)	0.482
LDL, mean (SD), (mg/dL)	2.97 (0.84)	3.1 (0.75)	0.853
UA, mean (SD), (mmol/dL)	375.7 (128.53)	334.27 (76.84)	0.443
SBP, mean (SD), (mmHg)	144 (8.72)	143.55 (10.61)	0.746
DBP, mean (SD), (mmHg)	93.1 (7.45)	92.64 (9.01)	0.932

BMI: body mass index; WC: waist circumference; FBG: fasting blood glucose; HbA1c: glycosylated hemoglobin; TG: triglyceride; TC: total cholesterol; HDL: high density lipoprotein; LDL: low density lipoprotein; UA: uric acid; SBP: systolic blood pressure; DBP: diastolic blood pressure.

**Table 2:** Simple parameters of body fat.

Variable	Group	2 weeks		4 weeks		8 weeks		12 weeks		3-month follow-up	
		Mean ± SD,P		Mean ± SD,P		Mean ± SD,P		Mean ± SD,P		Mean ± SD,P	
WC	Acupuncture	1.710 ± 1.312^*∗∗*^	≤0.001	2.350 ± 1.730^*∗∗*^	0.002	3.000 ± 2.475^*∗∗*^	0.001	4.000 ± 3.142^*∗∗*^	0.003	4.880 ± 3.967^*∗∗*^	0.002
	Sham	0.001 ± 1.155		0.690 ± 1.195		0.440 ± 1.315		0.870 ± 1.746^*∗*^		1.630 ± 3.722	
Weight	Acupuncture	0.91 ± 1.18^*∗∗*^	0.153	1.46 ± 1.53^*∗∗*^	0.058	2.42 ± 1.87^*∗∗*^	0.002	3.03 ± 2.75^*∗∗*^	0.011	4.00 ± 3.86^*∗∗*^	0.007
	Sham	0.762 ± 1.751^*∗∗*^		1.088 ± 2.714^*∗*^		0.988 ± 3.017		1.400 ± 3.524		1.638 ± 4.162	
BMI	Acupuncture	0.24 ± 0.437^*∗∗*^	0.158	0.470 ± 0.517^*∗∗*^	0.62	0.940 ± 0.748^*∗∗*^	0.001	1.060 ± 0.966^*∗∗*^	0.010	1.350 ± 1.169^*∗∗*^	0.007
	Sham	0.250 ± 0.775		0.380 ± 1.025^*∗∗*^		0.380 ± 1.258		0.440 ± 1.315		0.560 ± 1.590	
WHR	Acupuncture	0.010 ± 0.249	0.004	0.010 ± 0.021	0.032	0.016 ± 0.028^*∗*^	0.028	0.028 ± 0.035^*∗∗*^	0.004	0.024 ± 0.027^*∗∗*^	0.010
	Sham	−0.004 ± 0.021		0.001 ± 0.012		−0.001 ± 0.017		−0.001 ± 0.021		0.001 ± 0.022	
HC	Acupuncture	0.710 ± 2.365	0.384	1.240 ± 1.921^*∗*^	0.107	1.410 ± 2.063^*∗∗*^	0.085	1.060 ± 1.952^*∗∗*^	0.118	2.410 ± 2.740^*∗∗*^	0.054
	Sham	0.560 ± 1.825		0.560 ± 0.727^*∗∗*^		0.630 ± 0.957^*∗*^		0.880 ± 1.708^*∗*^		1.500 ± 2.221^*∗*^	

^*∗*^Within-group  *P* < 0.05 and ^*∗∗*^within-group  *P* < 0.01.

**Table 3:** Difference of the biochemical markers of patients with MetS treated with acupuncture and sham acupuncture.

Variable	Group	12 week	*P* value
FBG	Acupuncture	0.090 ± 1.245	0.217
	Sham	−0.272 ± 0.564^*∗*^	
HbA1c	Acupuncture	0.100 ± 0.333	0.008
	Sham	−1.019 ± 1.046^*∗*^	
TG	Acupuncture	0.617 ± 1.164	0.046
	Sham	−1.076 ± 2.285	
HDL	Acupuncture	−0.024 ± 0.211	0.814
	Sham	−0.043 ± 0.162	
LDL	Acupuncture	0.021 ± 0.364	0.477
	Sham	−0.304 ± 0.832	
TC	Acupuncture	0.019 ± 0.439	0.019
	Sham	0.919 ± 1.321	
UA	Acupuncture	48.500 ± 62.388^*∗*^	0.059
	Sham	−14.450 ± 45.735	
SBP	Acupuncture	9.900 ± 4.458^*∗∗*^	≤0.001
	Sham	−2.360 ± 5.278	
DBP	Acupuncture	6.200 ± 3.425^*∗∗*^	≤0.001
	Sham	−1.180 ± 1.940	

^*∗*^Within-group  *P* < 0.05 and^*∗∗*^within-group  *P* < 0.01.

## Additional Points

*Findings.* (1) The efficacy of acupuncture for the treatment of abdominal obesity was studied in a pilot study, Institute of Acupuncture and Moxibustion, China Academy of Chinese Medical Sciences, ZZ08012. (2) The evaluation of the prediabetic curative effect of acupuncture intervention and the management pattern research was based on the WeChat platform and the case registration system, State Administration of Traditional Chinese Medicine, JDZX2015137.
