# Supplementary material for: The Efficacy of Acupuncture on Anthropometric Measures and the Biochemical Markers for Metabolic Syndrome: A Randomized Controlled Pilot Study
Source: Evid Based Complement Alternat Med. 2017 Oct 31;2017:8598210. doi: 10.1155/2017/8598210 (PMC5684541; doi:10.1155/2017/8598210)
Supplement: Supplementary file 1 — Acupoints location and manipulation. [file 8598210.f1.doc]

Supplement Table Acupoints location and manipulation

|  | Acupuncture | | Shame acupuncture | |
| --- | --- | --- | --- | --- |
|  | location | manipulation | location | manipulation |
| Tianshu  (ST 25,double) | On the same level of the umbilicus,and 2cun lateral to the anterior midline. | perpendicular needling,  1.0-2.0cun | 0.5cm Beside ST 25 | perpendicular needling,  Pierce the skin |
| Zhongwan  (CV 12) | On the anterior midline, 4cun above the umbilicus. | perpendicular needling,  0.8-1.8cun | 0.5cm Beside CV 12 | perpendicular needling,  Pierce the skin |
| Xiawan  (CV 10) | On the anterior midline, 2cun above the umbilicus. | perpendicular needling,  1.0-2.0cun | 0.5cm Beside CV 10 | perpendicular needling,  Pierce the skin |
| Daheng  (SP 15,double) | On the same level of the umbilicus, 4 cun lateral to the anterior midline. | perpendicular needling,  1.0-2.0cun | 0.5cm Beside SP 15 | perpendicular needling,  Pierce the skin |
| Daimai  (GB 26,double) | when the eleventh rib free-end below vertical and umbilical horizontal line intersection point | perpendicular needling,  0.5-1.2cun | 0.5cm Beside GB 25 | perpendicular needling,  Pierce the skin |
| Liangmen  (ST 21,double) | On the same level of the CV 12,and 4cun lateral to the anterior midline. | perpendicular needling,  0.8-1.8cun | 0.5cm Beside ST 21 | perpendicular needling,  Pierce the skin |
| Qihai  (CV 6) | On the anterior midline, 1.5cun below the umbilicus. | perpendicular needling,  0.8-1.8cun | 0.5cm Beside CV 6 | perpendicular needling,  Pierce the skin |
| Zusanli  (ST 36,double) | 3cun directly below Dubi, and one finger-breadth lateral to the anterior border of the tibia. | perpendicular needling,  1.0-1.2cun,until the patients with strong sense of needle | 0.5cm Beside ST 36 | perpendicular needling,  Pierce the skin |
